# Chemically-stable flexible transparent electrode: gold-electrodeposited on embedded silver nanowires

**DOI:** 10.1038/s41598-023-44674-7

**Published:** 2023-10-16

**Authors:** Mostafa Gholami, Fariba Tajabadi, Nima Taghavinia, Alireza Moshfegh

**Affiliations:** 1 Department of Physics, Sharif University of Technology, Tehran, 11155-9161 Iran; 2 Department of Nanotechnology and Advanced Materials, Materials and Energy Research Center, PO Box 31787-316, Karaj, Iran; 3 Nano Center-Institute for Convergence Science and Technology, Sharif University of Technology, Tehran, 14588-8969 Iran

**Keywords:** Electronics, photonics and device physics, Nanowires

## Abstract

Silver nanowires (AgNWs) with a low diameter, high aspect ratio, stable suspension, and easy synthesis have recently attracted the optoelectronic industry as a low-cost alternative to indium tin oxide transparent conductive films. However, silver nanowires are not chemically stable, and their conductivity diminishes over time due to reactions with atmospheric components. This is a bottleneck for their wide industrial applications. In this study, we aim to address this issue by synthesizing silver nanowires with an average diameter of approximately 65 nm and a length of approximately 13 µm. The prepared Ag nanowires are then applied to fabricate transparent, flexible, and chemically stable conductive films. The fabrication includes spraying of silver nanowires suspension on a glass substrate followed by Dr. blade coating of polystyrene (PS) solution and delamination of the PS-AgNWs film. The resulting film exhibits an optimum sheet resistance of 24 Ω/□ and transmittance of 84%. To further enhance the stability of the transparent conductive film, the facial and scalable double pulse electrodeposition method is used for coating of gold on the exposed surface of the AgNWs embedded in PS. The final transparent film with gold coating demonstrates a remarkable stability under harsh conditions including long exposure to UV light and nitric acid solution. After 100 min of UV/Ozone treatment, the increase in sheet resistance of the optimal PS-AgNW@Au sample is 15.6 times lower than the samples without gold coating. In addition, the change in sheet resistance after 2000 bending cycles in the optimal PS-AgNW@Au electrode is measured and it showed an increase of only 22% of its initial sheet resistance indicating its good flexibility. The proposed electrode performs an excellent chemical stability, good conductivity, transparency, and flexibility that makes it a potential candidate for various optoelectronic devices.

## Introduction

Transparent conductive electrodes (TCE) are important components in optoelectronic devices such as touch screens, solar cells, organic light emitting diodes (OLEDs), and transparent heaters^1^. In most cases, indium tin oxide (ITO) is used as a TCE in optoelectronic devices because of its high transparency and low sheet resistance. However, the use of ITO faces several challenges such as UV radiation absorbance, indium scarcity, vacuum deposition method, and high cost^2^. Additionally, ITO is brittle due to its ceramic nature, and its electrical properties deteriorate with bending, so it cannot be perfect for flexible optoelectronics^3^. Other transparent conductive oxides^4,5^ such as fluorine-doped tin oxide (FTO)^6^ and ZnO: Al (AZO)^7^, carbon nanotubes^8^, graphene^9^, conductive polymer^10^, metal nanowires^11^, etc. can be considered as alternatives for ITO. But carbon nanotube or conductive polymer based TCE electrodes suffer from high cost while metal oxide based TCE suffer from low flexibility. Thus, there is a challenge to overcome these barriers.

Electrically, silver metal is the most conductive element with electrical conductivity of 6.3 × 10^7^ Sm^−1^^12^. Among the alternative shapes, silver nanowires (AgNWs) gained attention due to their special optoelectronic properties, which are better than their bulk performance. Some practical features of AgNWs are excellent electrical conductivity, tunable optical properties and high light transmission, solution-based and scalable processing, and flexibility^13^. AgNWs have been used in various applications including light-emitting diodes^14,15^, solar cells^16,17^, wearable electronic devices^18,19^, flexible electronics^20,21^, touch screens^22^, heaters^23^. However, AgNWs as TCEs have disadvantages such as limited long-term stability, high surface roughness (due to the overlapping of nanowires)^24^, weak adhesion to the substrate, poor contact at wire-wire junctions. Furthermore, AgNWs exposed to ambient air undergo chemical corrosion due to surface interaction with atmospheric components which leads to diminish in their electrical conductivity over time. It is reported that sulfidation is the main corrosion of AgNWs in the air^25^. In perovskite solar cells application, halide ions react with silver causing an increase in the resistance of nanowires^26^. The instability problem of AgNWs is still a major challenge that requires further studies to improve their stability^27^.

Semi-embedding of AgNWs in polymer surfaces has been shown to reduce surface roughness, improve adhesion to the substrate, and increase the stability of the nanowires^28^. Various polymers^29^ such as colorless polyimide^28^, hydrogen bonds-based polyimide^30^, UV-curable epoxy^31^ polymethyl methacrylate (PMMA)^32^, chitosan biopolymer hybrid^33^, glass-fabric reinforced transparent composite (GFRHybrimer) film^34^ have been used for semi-embedding of AgNWs. Although all of these AgNWs-based TCE exhibits good conductivity, flexibility and adhesion, but they were produced by lab-scale spin coating method. In this research, we utilized a scalable spray deposition method for depositing AgNWs film.

To address the stability issue of AgNWs, protective coatings have been used in previous studies. These coatings include metal oxides (MoO_x_^35^ WO_x_^36^ NiO^37^ ZnO and Al_2_O_3_^38^), metals (palladium (Pd)^39^, nickel (Ni)^26^ gold (Au)^40–42^), graphene^43,44^, and polymers. Though these coatings can improve chemical stability, they may have negative effects on optoelectronic properties due to their low electrical conductivity like most metal oxides and polymers. Additionally, some coatings like ZnO have limited stability in different media^45^. Among different protective coatings, gold has excellent chemical stability, high electrical conductivity, good lattice matching with silver, and can be easily deposited on the surface of AgNWs. The gold protective layer is selectively deposited only on the surface of the AgNWs film, not on the surface of the polymer matrix. Therefore, the optical properties of the electrode do not change significantly. Chen et al. reported that gold nanoparticles were loaded onto the surface of AgNWs by immersing the AgNWs film in an aqueous solution containing dispersed AuNPs for 1 min. A sudden increase in the electrical resistance of the nanowires (thermal stability criterion) was observed for AgNW@AuNP films at around 300 °C and for AgNWs at 200 °C. This result indicates an improvement in the thermal stability of the electrode. SEM images showed that gold nanoparticles were scattered on the surface of the nanowires, but did not completely coat them^41^. In a report published in Nature Nanotechnology (Choi et al.), gold was deposited on the surface of AgNWs using sodium sulfite (galvanic-free deposition). Investigation into treatments with 1.5M hydrogen peroxide (H_2_O_2_) (as the oxidant) and ultraviolet/ozone (UV/O_3_) (ozone as oxidant) showed that the stability of the nanowires increased with gold deposition^39^. Despite the publication of such reports, electrodeposition of gold on imbedded AgNWs-polymer film has not been reported.

In this research, we utilized a scalable spray deposition method for depositing AgNWs film^46,47^. We have selected electrodeposition method as a controllable method for deposition of gold on AgNWs imbedded in polystyrene film to improve chemical stability of final TCE electrode. The stability of the samples was examined under severe oxidation in the UV/O_3_ treatment and in the corrosive environment of nitric acid. In addition, physical properties such as optical transparency, flexibility, and adhesion of AgNWs to the surface of the fabricated electrode were also examined.

## Experimental

### Materials

All materials were used as received without further purification. silver nitrate (AgNO_3_, 99.8%) and ethylene glycol (C_2_H_6_O_2_, 99.9%) was purchased from Merck. Iron (III) chloride hexahydrate (FeCl_3_.6H_2_O, 98%), copper (II) chloride (CuCl_2_, 98%) and polyvinylpyrrolidone (PVP) were obtained from Sigma Aldrich. HAuCl_4_ solution (0.1mM) was prepared from IRASOL.

### Silver nanowires synthesis

AgNWs have been synthesized using a so-called polyol process^48^. Silver nitrate (0.11 mM) was dissolved in 24 mL of ethylene glycol (EG). The second solution contains two types of polyvinyl pyrrolidone (PVP) with molecular `mass 40,000 (concentration 5 mg/mL) and 360,000 (concentration 10 mg/mL), dissolved in 24 mL of EG. The solutions were then combined. Finally, a solution of CuCl_2_ (0.0003 g) and FeCl_3_.6H_2_O (0.0006 g) dissolved in a 1 mL of EG were added. Less than 90 s later, the solution was poured into a reaction vessel at 150 °C and remained at that temperature for 7 h. After cooling to room temperature, ethanol was added and the mixture was centrifuged twice in two steps (4500 rpm, 4 min and 3800 rpm, 4 min) to separate the AgNWs. The final dispersion of the AgNWs in ethanol (1 mg/mL) was stored at 4 °C for future use.

### Fabrication of TCE

**Figure 1:**
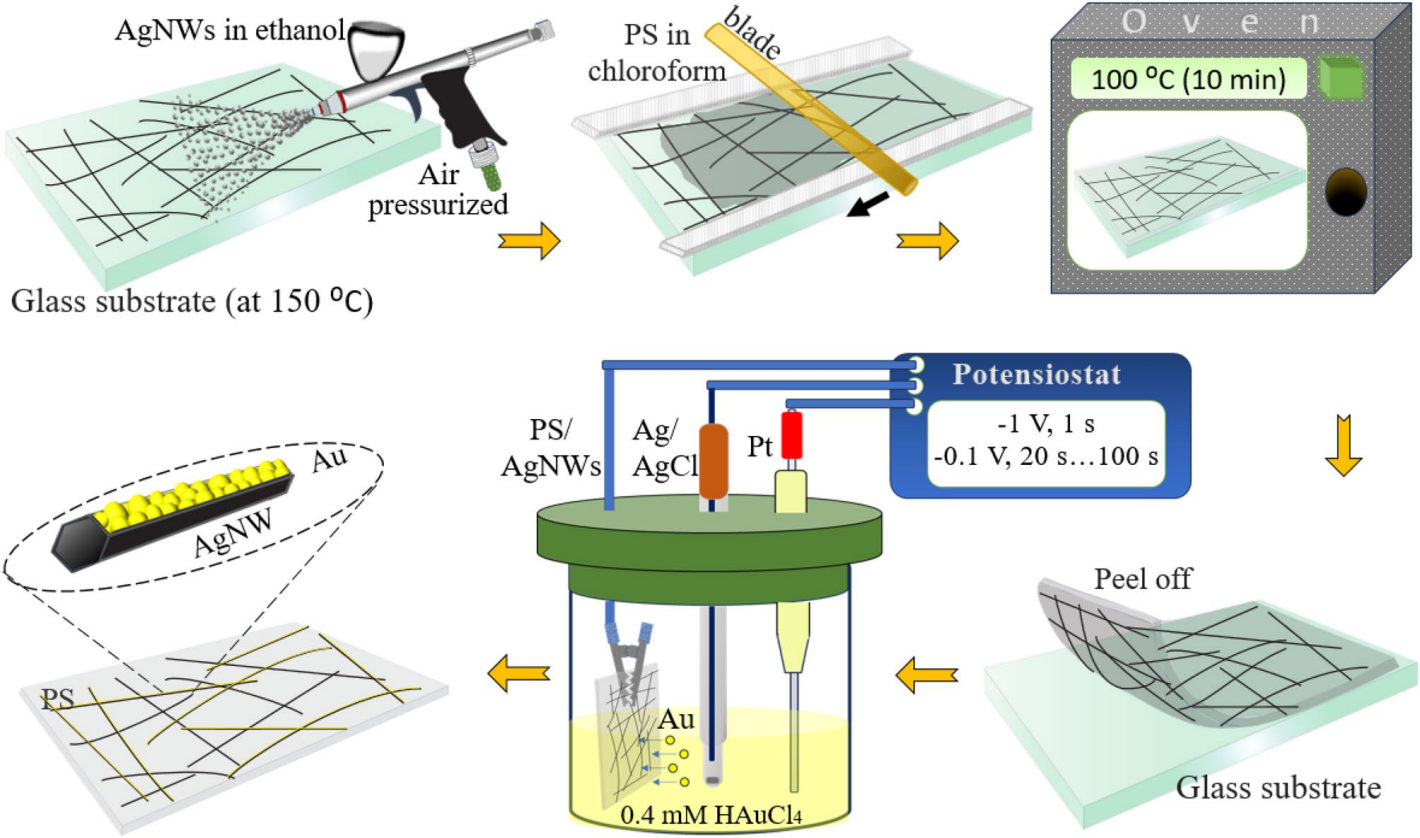
Schematic illustration of preparation of PS-AgNWs electrode and Au coating.

As shown in Fig. 1, AgNWs with a concentration of 0.5 mg/mL were sprayed on glass substrate (pre-cleaned with DI water, ethanol, acetone and followed by UV-Ozone cleaner) with handheld commercial airbrush spray gun. The preset pressure of airbrush was set at 3 bar. During spraying the substrate (5 × 10 cm^2^) was maintained at a constant temperature of 150 °C. AgNWs Films with different sheet resistances and transparency could be obtained by adjusting the amount of solution sprayed.

For fabrication of PS-AgNWs films, a solution of PS in chloroform (0.2 mg/mL) was deposited on the glass-AgNWs film by Dr. Blade method and dried at room temperature for 20 min. Then the samples were placed in an oven at 100 °C for 10 min. Finally, the prepared PS-AgNWs film was peeled off after cooling to room temperature.

### Gold electrodeposition

A three electrode (Sharif Solar Co.) electrochemical cell with Pt as counter electrode, Ag/AgCl as reference electrode and PS-AgNWs as the working electrode was used for deposition gold on the prepared AgNWs in HAuCl_4_ aqueous solution (0.4 mM) by potentiostat/galvanostatic instrument (Sharif Solar Co.) device (Fig. 1). A double-potential-pulse electrodeposition method was, including E_1_ = − 1 V (nucleation step) for 1 s and E_2_ = − 0.1 V (growth step) for 20 s, 40 s, 60 s, and 80 s^49,50^. The solution was rotating for 1 s. Gold nanoparticle layers with different thicknesses were made by changing the growth step time.

### Characterization

The morphology and elemental composition of the prepared electrocatalysts were explored by field-emission scanning electron microscopy (FESEM) and energy-dispersive X-ray emission spectroscopy (EDX) on Tescan FE-SEM MIRA3 with an acceleration voltage of 15 kV. Atomic force microscopy (AFM) technique (AutoProbe Cp-Research Veeco) in non-contact mode was also used to describe the surface roughness. The optical transparency of the samples was investigated by an Avantes spectrometer (AvaSpec-USL2048TEC) in a range of UV–Visible wavelengths. To analyze the surface chemical composition of the prepared electrocatalysts, X-ray photoelectron spectroscopy (XPS) measurements were performed using BesTec (Germany) which utilizes an Al-Kα X-ray source with energy at 1486.6 eV. The samples were introduced into the ultrahigh vacuum (UHV) chamber and then evacuated to a base pressure of ~ 10^−10^ mbar. All obtained binding energy values were calibrated by fixing the C (1s) core level peak at 284.8 eV with an energy resolution of ± 0.1 eV. Finally, the characteristic peaks in the high-resolution XPS spectra were deconvoluted for quantitative surface chemical analysis. The sheet resistance of the samples was measured by four-point probe instrument (Keithley 2500).

## Results and discussion

### Synthesis and morphology

**Figure 2:**
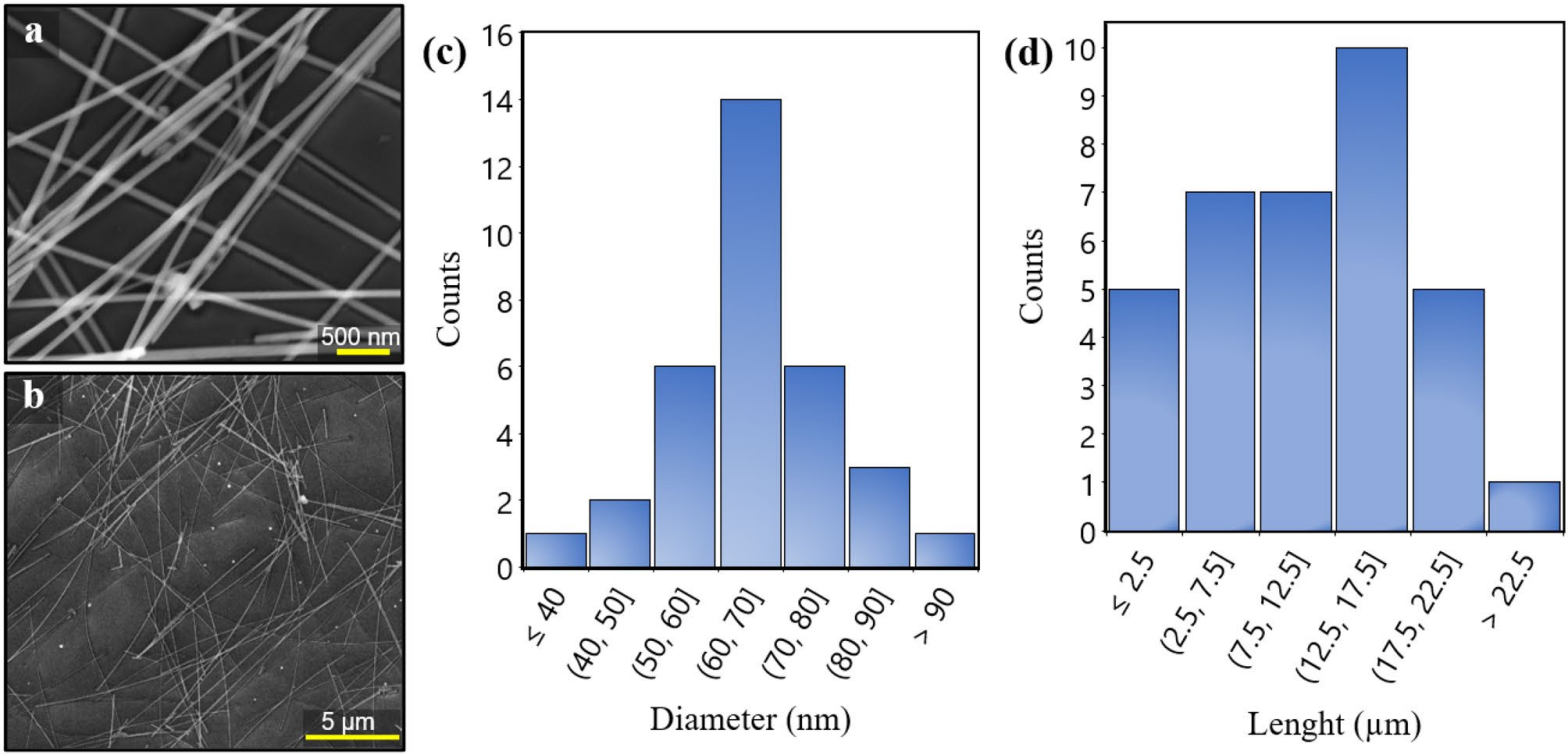
(**a**), (**b**) FESEM image of AgNWs deposited by drop cast method on glass, (**c**), (**d)** Diameter and length of AgNWs in FESEM images measured using ImageJ software.

Morphology of the prepared AgNWs is studied by FESEM analysis. As shown in Fig. 2, AgNWs shows average diameters ~ 65 nm and length about 13 µm, indicating a high aspect ratio of about 200.

For obtaining a uniform network, AgNWs were deposited on the glass surface by spray method under optimized conditions. The effect of the substrate temperature during spraying on the sheet resistance of the coated nanowire network was investigated, which is shown in Fig. S1. As seen in this figure, higher substrate temperature results in a lower sheet resistance of the AgNWs. However, the resistance reduction is relatively small so we selected a moderate temperature of 150°C during spray of AgNWs suspension. A similar result has been also reported for the transparent film of nanowires deposited by the spray method^46^ that is due to partially fused junction of silver nanowires deposited in this approach. The same electrical resistance (See Table S1) for the AgNWs network on glass substrate and AgNWs embedded in polystyrene film indicates a complete transfer of the nanowires from the glass surface to the polymer matrix, resulting in a uniform PS-AgNWs film with a thickness of ~ 30 µm. As expected, the PS surface containing embedded AgNWs is conductive, while the opposite surface is non-conductive. In addition, the most of the AgNWs are entrapped inside the polystyrene film as confirmed by AFM images. Based on AFM data analysis the surface roughness of the films decreased from about 61.0 nm for the AgNW network on glass to about 3.0 nm for PS-AgNW (Fig. S5). So, they are not exposed to the environment and the polystyrene film acts as a protective layer. A relatively small part of the AgNWs surface is exposed to the environment and needs to be protected from chemical reactions. By applying a suitable potential, this exposed part can be covered with a gold film.

We first studied galvanic replacement method for coating gold on the AgNWs surface in 0.4 mM of aqueous HAuCl_4_ solution. Electrode resistance increase with longer reaction times, indicating a change in the structure of the nanowires (Fig. S2). The change in the structure of the nanowires and eventual breaking of the nanowires has been reported previously^51^. Due to the poor results of galvanic replacement for gold deposition on AgNWs surface, we switched to the electrodeposition method as a more controllable deposition method. Additionally, we selected the double-potential-pulse electrodeposition method to form a more uniform coating of gold nanoparticles, according to previous reports^49,50^. In order to choose the best potentials for gold deposition, the voltammograms of PS-AgNWs and FTO electrodes were recorded in a 0.4 mM aqueous HAuCl_4_ solution, as shown in Fig. S3. Using the AgNWs electrode, the reduction peak of gold could not be determined because the reduction peak of gold was within the limit of the oxidation peak of silver. Therefore, in this experiment, the FTO electrode was used as the working electrode. The reduction peak of gold was seen at E = + 0.51 V, which was in good agreement with previous reports^52^. Based on the peak of silver oxidation, we had to choose the applied potentials with a distance from the starting point of silver oxidation (E1 = − 1 V for 1 s and E2 = − 0.1 V 20 s, 40 s, 60 s, 80 s).

**Figure 3:**
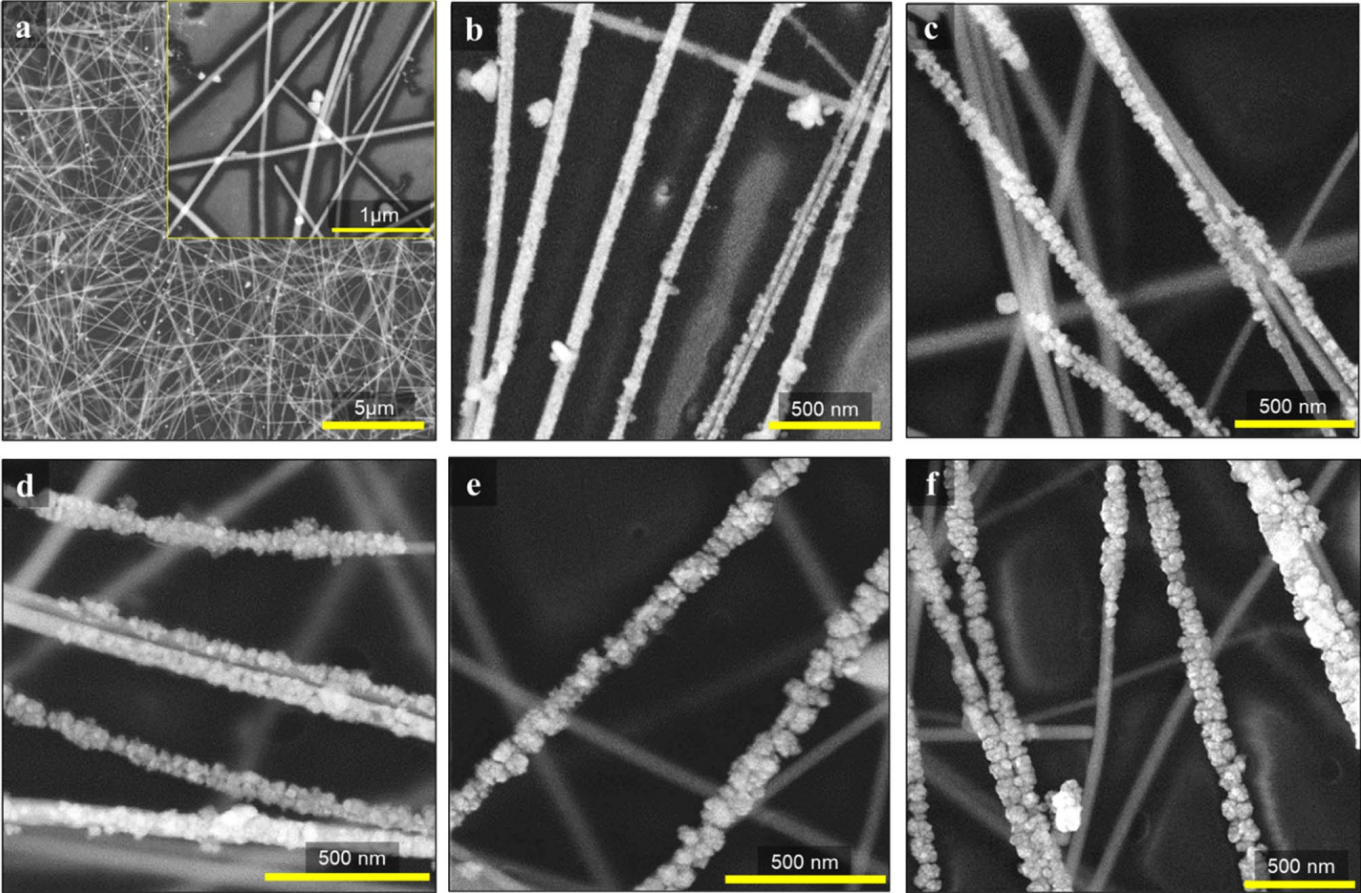
FE-SEM images of: (**a**) PS-AgNW, (**b**) PS-AgNW@Au(1 s,1 V) (nucleation pulse in the gold plating process), (**c**) PS-AgNW@Au(20), (**d**) PS-AgNW@Au(40) and (**e**) PS-AgNW@Au(60) (**f**) PS-AgNW@Au(80).

The FE-SEM image of the PS-AgNW and different PS-AgNW@Au films can be seen in Fig. 3. Ag NWs show a smooth surface with diameter 65 nm like AgNWs network on glass substrate. after applying of growth pulse (− 1 V, 1 s) a uniform coating of small gold nanoparticles is formed on the AgNWs surface which is exposed to the electrolyte solution, as shown in Fig. 3b. Figure 3c–f shows FE-SEM images for different times of growth pulse (− 0.1 V), which result in a higher amount of Au deposition with larger size on the AgNW-PS surface. With a longer growth time step, the size of the gold nanoparticles increases, leading to their connection and the formation of a nearly complete coating of gold on the AgNWs. So that after 60 s electrodeposition time, the diameter of the outer nanowires increases from 65 to 94 nm. It should be mentioned that Au only deposits on the surface of AgNWs that are in contact with air, so the totally embedded AgNWs in the PS film do not have Au coating. The sheet resistance of the AgNWs network (sprayed) in in Fig. 3 is measured approximately 15 Ω/□.

The energy dispersive X-ray analysis (EDX analysis) of the PS-AgNW@Au(40) and PS-AgNW@Au(80) samples is shown in Fig. S4. The presence of gold in the EDX results confirms that gold has been coated onto the surface of the nanowires using the electrodeposition method. In the PS-AgNW@Au(40) sample, the atomic percentage of silver and gold is 1% and 0.4%, respectively. In the PS-AgNW@Au(80) sample, compared to the PS-AgNW@Au(40) sample the percentage of gold (1.1%) has increased, as confirmed by FESEM analysis.

According to AFM image analysis (Fig. S5), the root mean square (RMS) surface roughness of the PS-AgNW@Au(60) is about 36.3 nm that is lower than the RMS surface roughness of the silver nanowires network formed on the glass substrate ~ 61 nm.

### Transmittance and surface chemical composition

**Figure 4:**
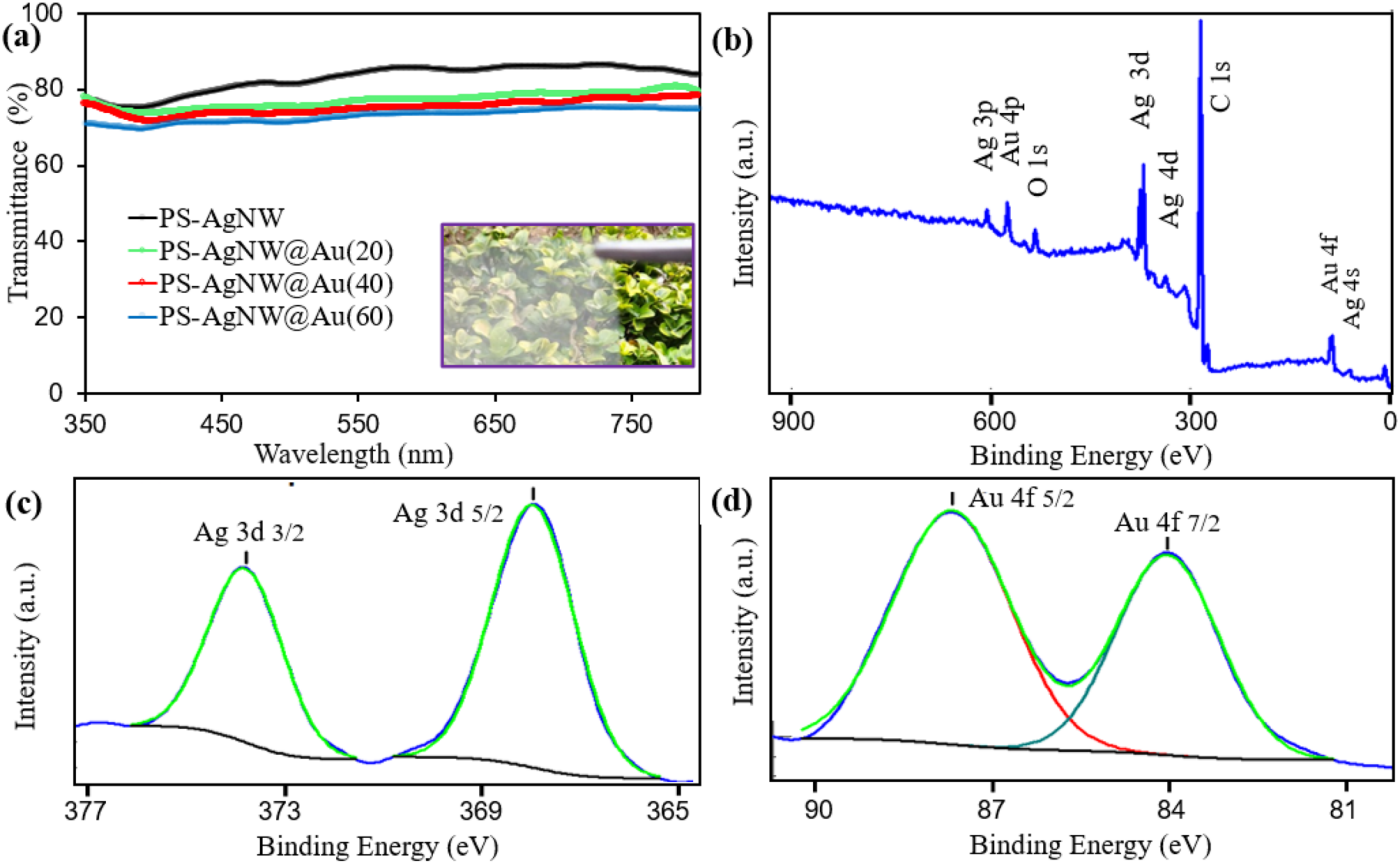
(**a**) Transmittance of PS-AgNW and PS-AgNW@Au(n) samples in visible region. (**b**) XPS survey spectrum of the PS-AgNW@Au(40) sample, and high-resolution spectra of (**c**) Ag(3d) and (**d**) Au(4f).

The transmittance of PS-AgNW and different PS-AgNW@Au films are reported in Fig. 4a. The light transmission of the PS-AgNW sample (with sheet resistance = 24 Ω/□) is in the range of 400 to 800 nm was measured to be about 83.9%. The AgNWs network has a higher transmittance, (~ 88%) and the polymer substrate reduces the transparency of the samples by about 4%. The deposition of gold on the external surface of AgNWs decreases the transparency of 6.8%, 8.7%, and 10.6% for the PS-AgNW@Au(20), PS-AgNW@Au(40) and PS-AgNW@Au(60) samples, respectively. As the growth pulse time increases, more gold is electrodeposited, resulting in decreased transparency. This is a logical result that can be expected due to the increase in the diameter of the nanowires observed in FE-SEM images.

The XPS survey spectrum of PS-AgNW@Au(40) sample (Fig. 4b) shows the presence of C, O, Ag and Au elements on the surface of the sample. The carbon element has the largest peak, and almost 90% of the surface elements are carbon. Considering that most of the surface is composed of PS and there are always surface pollutants, this issue is justified. Figure 4c,d show the high-resolution XPS spectra of the Ag(3d) and Au(4f) core level surface peaks, respectively. The Ag(3d) spectrum contains two peaks at 367.8 eV for the Ag(3d_5/2_) and other at 373.8 eV for the Ag(3d_3/2_), which is in good agreement with the published values of Ag(3d)^53,54^.

All spectra of the Ag(3d) and Au(4f) were deconvoluted with one peak, which indicates that all silver and gold are in metallic state. For the Au(4f), there are two peaks measured at 84.0 and 87.7 eV for the Au(4f_7/2_) and Au(4f_5/2_), respectively. These results are in good agreement with the recent published reports^53,54^. After analysis of the area surrounded by each peak, the atomic surface concentrations of Ag and Au were determined to be about 4.4% and 1.3%, respectively.

Considering that silver and gold are completely in metallic state, the presence of elemental oxygen on the surface is also due to bonding with carbon atom. According to our XPS data analysis, it was found that the gold element is deposited on the surface of the nanowires, and in the PS-AgNW@Au(40) sample. It should be noted that gold covers parts of the surface of the nanowires, which is in agreement with the FESEM observation.

### Stability test

**Figure 5:**
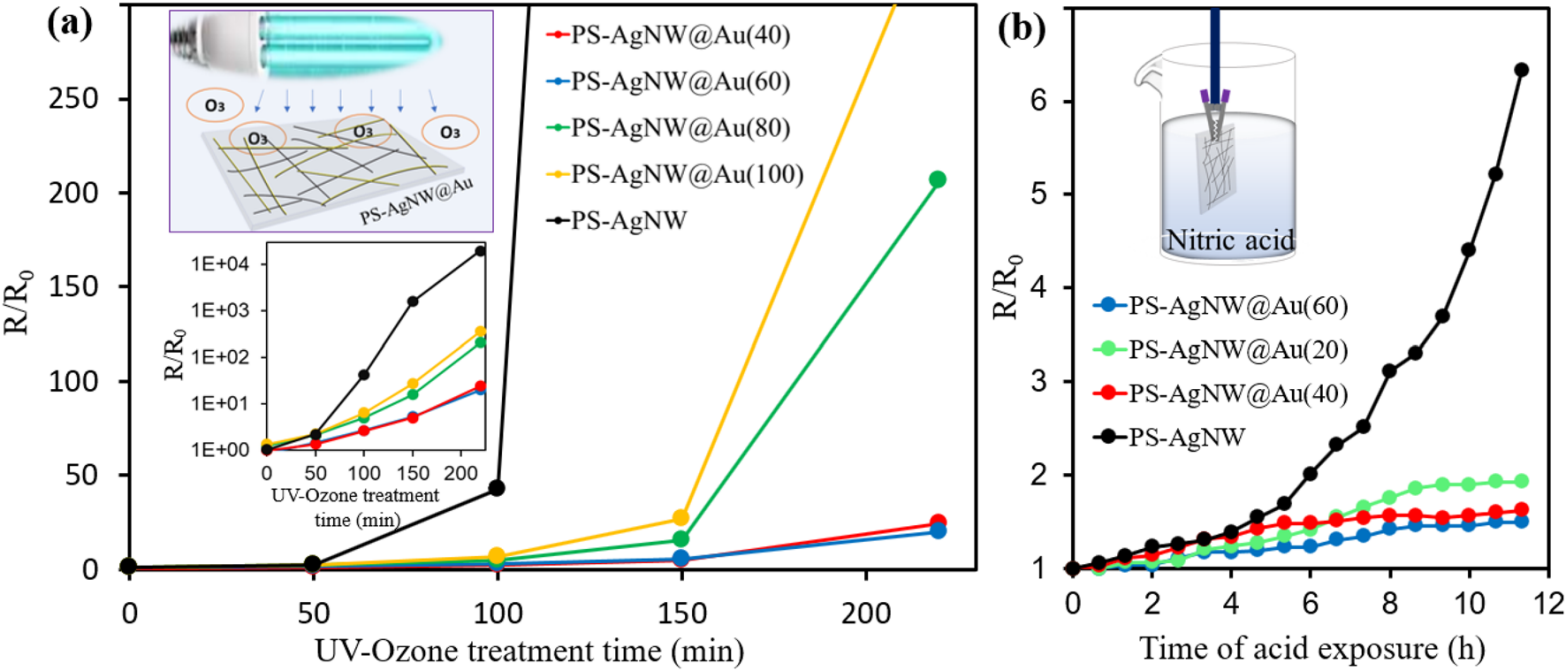
Stability evaluation of different samples exposed to: (**a**) UV-Ozone lamps. (**b**) diluted nitric acid solution. The change in relative sheet resistance (R/R_0_) as a function of exposure time for different samples. (**a**) Inset: Semi-logarithmic diagram of the changes with UV-Ozone duration time.

To confirm the stability of the prepared transparent conductive electrode, ultraviolet/ozone (UV/O_3_) treatment was performed. With the help of UV/O_3_ treatment, the rate of oxidation of the samples increases and it is expected that the samples will be destroyed after a short time. The degradation process of the samples was investigated by measuring the sheet resistance of the samples over time. Different samples were fabricated at different times of gold deposition (40, 60, 80, 100 s), and the average results are shown in the diagram of Fig. 5a. The resistance of the PS-AgNW sample increased by 2.2 times after 50 min exposure to UV light. While the resistance change in gold-deposited samples was insignificant. After 100 min UV/O_3_ treatment, the increase in resistance in the PS-AgNW sample (R/R_0_ = 42) was much higher (15.5 times) than the changes in the resistance of the PS-AgNW@Au(60). We found that when the exposure time increases for longer times, the degradation of unprotected AgNWs occurs faster compared to gold-coated samples.

Then the samples were subjected to UV/O_3_ treatment for 150 and 220 min (from the beginning of the UV/O_3_ treatment process). The network of nanowires collapses at a certain time, and the increase in sheet resistance of electrode is remarkable. According to the stability diagram of the samples, the network collapse time for the PS-AgNW sample is earlier than all other samples, and all the PS-AgNW@Au(n) samples showed more stability than the PS-AgNW sample. During 220 min, the resistance change in the PS-AgNW@Au(60) sample was several times lower than that of the PS-AgNW sample. In the inner figure, the changes in the sheet resistance of the samples are drawn in a semi-logarithmic form so that variations in higher range of sheet resistances can also be seen. Although the gold coating in all the samples lead to more stability against oxidation in the UV/O_3_ environment, longer growth times more than 60s for gold electrodeposition result in lower stability relative to growth times of 40 and 60s.

In addition to stability against UV/O_3_, stability in nitric acid solution (5% by volume) was also investigated. The transparent electrodes are dipped into a very dilute nitric acid solution, and electrical resistances were measured every 40 min, and the results are displayed in Fig. 5b. Nitric acid can corrode silver but has no effect on gold^55^. If the resistance of the sample does not change or changes slowly, it indicates that there is a suitable coating of gold on the surface of the AgNWs. For the PS-AgNW sample, the electrical resistance after 11 h of dipping the sample in nitric acid solution increased to more than 6 times, while the resistance of all electrodes coated with gold changed to less than 2 times. These results confirm that the presence of gold coating improved the chemical stability of conductive electrodes.

### Flexibility and adhesion test

**Figure 6:**
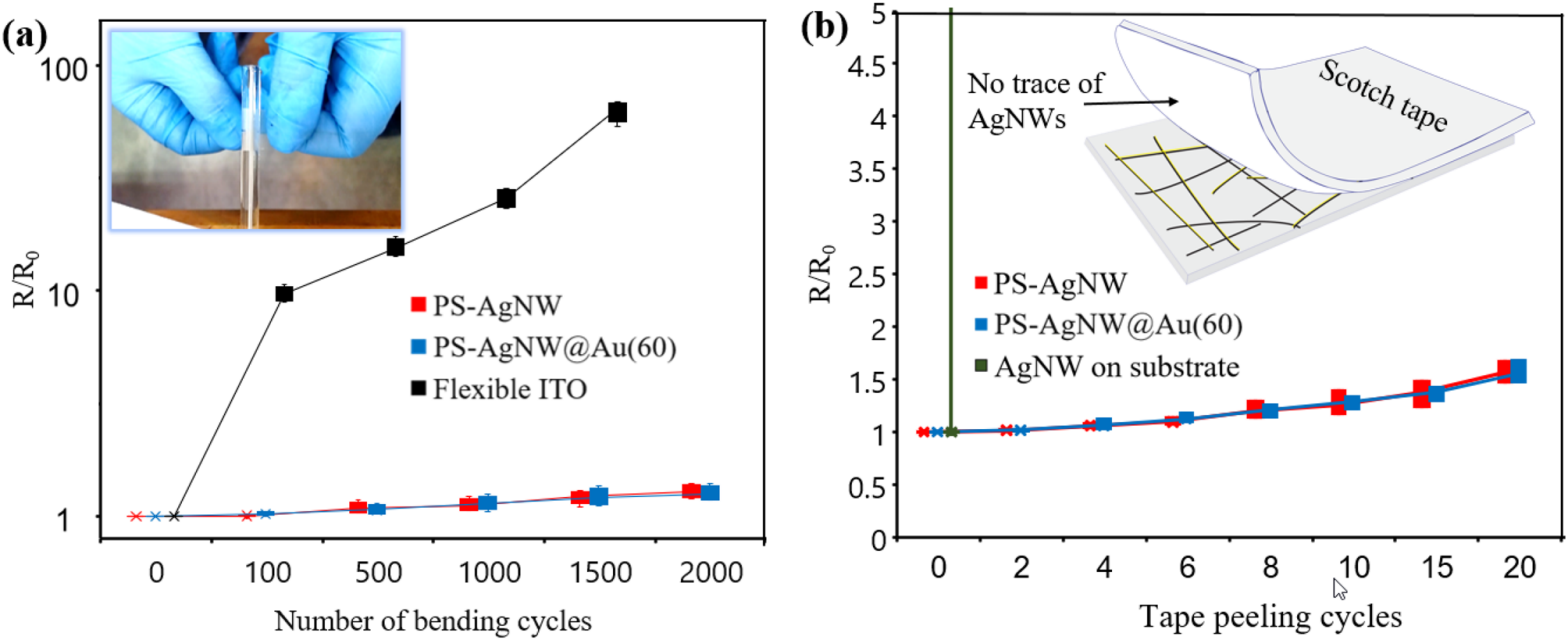
Flexibility and adhesion to substate of electrodes (**a**) Relative resistance changes caused by tape peeling cycles for ITO, PS-AgNW and PS-AgNW@Au(60) (**b**) Relative resistance changes caused by bending cycles for flexible ITO, PS-AgNW and PS-AgNW@Au(60).

Mechanical flexibility is one of the key advantages of AgNWs network-based electrodes. Flexibility plays an important role in applications such as wearable sensors, stretchable displays, and in general soft electronics. To examine the flexibility, the fabricated electrodes of PS-AgNW, PS-AgNW@Au(60), and flexible ITO electrode were tested under bending at a radius of 4 mm. Three electrodes for each mode (9 electrodes in total) were subjected to cycles of bending from 100 to 2000 loads. The results are presented in Fig. 6a using box and whisker diagrams (drawn in semi-logarithmic form). R_0_ represents the initial measured sheet resistance, and R represents the measured sheet resistance after internal bending.

For flexible ITO electrode, the sheet resistance increased almost 10 times after 100 cycles of bending. However, for the PS-AgNW and PS-AgNW@Au(60) samples, the change in sheet resistance was very small, with an average increase of only 2%. The drastic difference was observed for the ITO electrode compared to both PS-AgNW (three samples) and PS-AgNW@Au(60) electrodes at a higher numbers of bending cycles. After 1500 cycles of bending, the average sheet resistance for the flexible ITO increased by more than 60 times compared to the initial resistance, while for the PS-AgNW and PS-AgNW@Au(60) samples, the average resistance increased by 25%. There was no significant difference between the PS-AgNW and PS-AgNW@Au(60) electrodes in terms of flexibility. These results demonstrate that the electrodes prepared using this method exhibit excellent flexibility compared to flexible ITO. The observed excellent flexibility can be attributed to the mechanical stability of the nanowires in the polymer substrate, in addition to the inherent properties of the AgNWs. Additionally, the chemical stability of the gold-coated Ag nanowires did not affect the mechanical flexibility of the electrode. In a study by Wang et al.^56^ nickel coating (prepared by electroplating) was used to improve the stability of AgNWs. In this report, the bare nanowire network electrode failed the mechanical flexibility test after 600 cycles. By applying a nickel coating, the flexibility performance of the samples was much improved. It is worth noting that an initial increase in sheet resistance (approximately 2 times) was observed after the first 10,000 cycles in their study, which was not observed in the PS-AgNW and PS-AgNW@Au(60) samples.

AgNWs have very poor adhesion to the substrate (such as polymer or glass). An additional process is required to improve this adhesion^36^. To assess the adhesion of AgNWs to the substrate, a scotch tape test was performed on the fabricated electrodes^57,58^. In this report, a strip of Scotch tape was placed on the surface of the electrode and pressed with the help of a roller to completely stick to the surface. It is then peeled off. The sheet resistance variations were measured to investigate the effect of the scotch tape test on the nanowires network. Obviously, for the nanowires deposited on the substrate with the first test, the resistance tends to infinity. For PS-AgNW and PS-AgNW@Au(60) electrodes, tests were taken from six samples (three samples for each mode) and the box and whisker diagrams of the results are reported in Fig. 6b. In the case of the PS-AgNW electrode, after performing the test twice, the average sheet resistance of the electrode did not change significantly (less than 2%). Even after performing the scotch tape test 20 times, the sheet resistance of the electrode increases by almost 1.5 times, which is considered a small change in the type of test. This test was also performed for the PS-AgNW@Au(60) electrode. Its results were not significantly different from the PS-AgNW electrode. No traces of AgNWs were observed in the Scotch tape separated from the surface of these electrodes The results, shown in Fig. 6b, indicate that that the AgNWs and gold nanoparticles on it are well-adhered and mechanically stabilized in the polystyrene substrate.

## Conclusions

AgNWs with an average length of 13.0 µm and an average diameter of 65 nm were synthesized using the polyol method. The transparent and flexible conductive electrode was created by embedding silver nanowires on the surface of polystyrene and applying gold electrodeposition on the outer nanowires for better stabilization. XPS results for the PS-AgNW@Au(40) sample confirmed the presence of a gold coating on the surface of the AgNWs. The transparency of the PS-AgNW sample, with a sheet resistance of 24 Ω/□, was measured to be 83.9%. With the addition of a gold coating in the PS-AgNW@Au(20-40-60) samples, the transparency decreased in a range of 6.8 to 10.6%. The stability of the samples was tested in UV-Ozone and nitric acid environments, and it was found that the samples coated by gold showed higher stability. After 100 min of UV-Ozone treatment, the sheet resistance of the PS-AgNW sample increased by 42 times. In contrast, the optimal PS-AgNW@Au(60) sample only experienced a 2.6-fold increase, indicating a significant improvement in stability. The issue of adhesion between AgNWs and the substrate was successfully addressed in both the PS-AgNW and PS-AgNW@Au(n) samples. Furthermore, the flexibility of these samples was found to be superior to that of commercial flexible ITO electrodes.

### Supplementary Information

Supplementary Information.

## Data Availability

All data generated or analysed during this study are included in this published article (and its Supplementary Information file). Further inquiries can be directed to the corresponding authors.
