# Supplementary material for: Chemically-stable flexible transparent electrode: gold-electrodeposited on embedded silver nanowires
Source: Sci Rep. 2023 Oct 16;13:17511. doi: 10.1038/s41598-023-44674-7 (PMC10579339; doi:10.1038/s41598-023-44674-7)
Supplement: Supplementary file 1 — Supplementary Information. [file 41598_2023_44674_MOESM1_ESM.docx]

**Supplementary information**

**Chemically-stable flexible transparent electrode: Gold-electrodeposited on embedded silver nanowires**

Mostafa Gholami^1^, Fariba Tajabadi^2^, Nima Taghavinia^1,3*^, Alireza Moshfegh^1,3*^

^1^ Department of Physics, Sharif University of Technology, Tehran 11155-9161, Iran

^2^ Department of Nanotechnology and Advanced Materials, Materials and Energy Research Center, Karaj, PO Box 31787‑316, Iran.

^3^ Institute for Convergence Sci. and Technol., Sharif University of Technology, Tehran 14588-8969, Iran

*Corresponding authors: [taghavinia@sharif.edu](mailto:taghavinia@sharif.edu) and [moshfegh@sharif.edu](mailto:moshfegh@sharif.edu)

**Temperature dependence on sheet resistance**

During the spraying process, the glass substrates were subjected to different temperatures to investigate the effect of substrate heating on the sheet resistance of the silver nanowires (AgNWs). Figure S1 shows the measured resistance of nanowires sprayed on the substrate at different temperatures of 110, 150, and 190 °C with corresponding transparency. The degree of transparency of the samples is proportional to the density of loaded nanowires formed on the substrate surface. The results showed as the substrate temperature increased, the sheet resistance decreased. However, the decrease in the resistance was not significant and we found an average reduction of 23% during temperature rise from 110 to 190 °C. Therefore, higher temperatures were not used in the subsequent experiments, and all samples were sprayed at temperature of 150 °C.


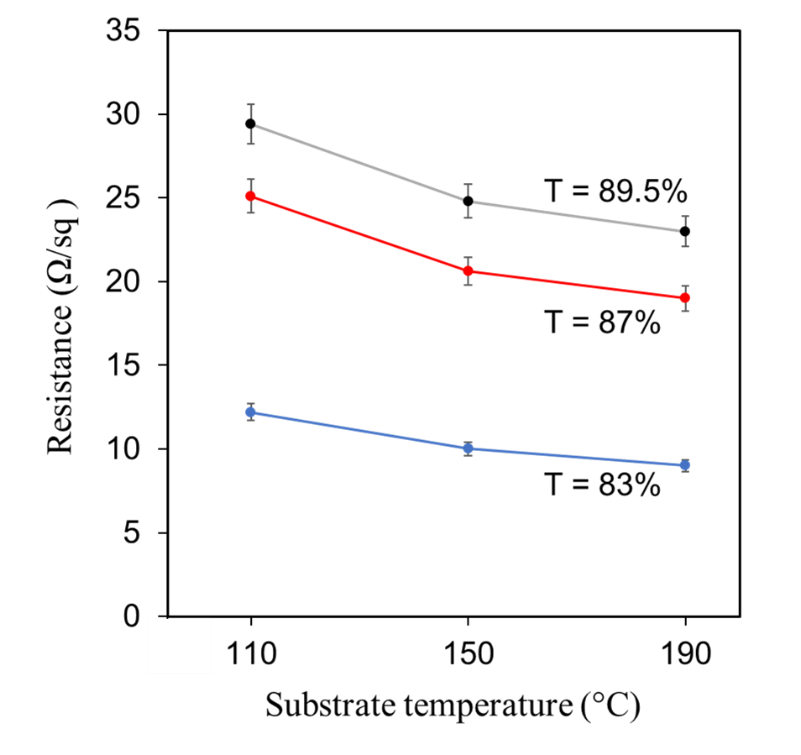


Figure S1. The effect of heating temperature on the sheet resistance of AgNWs network deposited on glass substrate along with corresponding transparency.

Table S1. Comparison of sheet resistances of AgNWs network on glass substrate and after embedding in polymer substrate

| Sheet resistance of AgNWs embedded in PS film (Ω/□) | Sheet resistance of AgNWs network on glass substrate (Ω/□) | Sample |
| --- | --- | --- |
| 16.2 | 16.1 | 1 |
| 15.4 | 15.3 | 2 |
| 14.2 | 14.2 | 3 |

**Gold deposition on AgNWs**

To deposit gold onto the AgNWs, the electrode was first immersed in a HAuCl4 solution to replace silver atoms with gold atoms through a galvanic replacement method. As shown in Fig. S2, the sheet resistance of the electrode initially increased due to the destruction of the nanowires. However, by applying a voltage (electrodeposition method), gold ions were regenerated and deposited onto the surface of the nanowires, preventing further destruction of samples. Silver atoms are not separated from the surface of the nanowire. Thus, as a result, the sheet resistance of the nanowires did not change significantly even after 100 seconds of deposition.


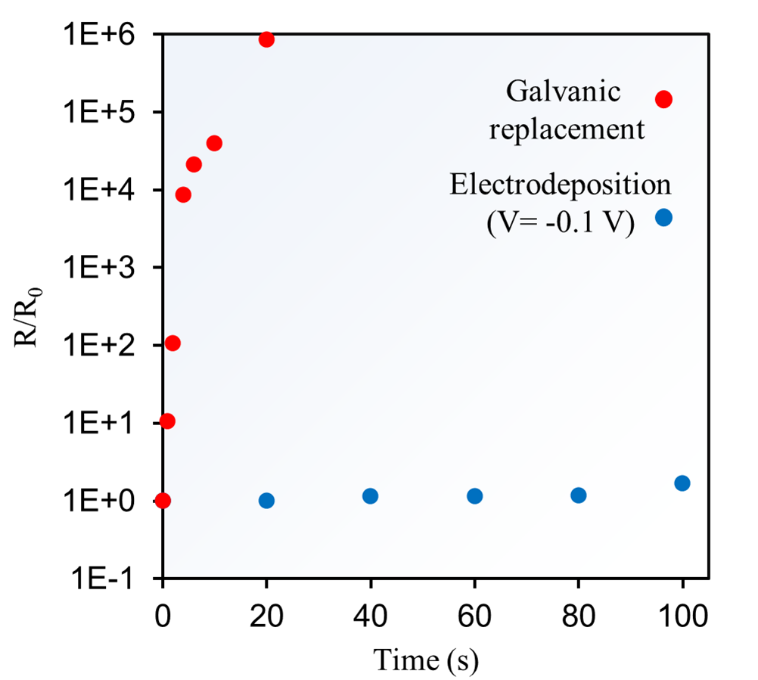


Figure S2. Relative sheet resistance changes of AgNWs in HAuCl_4_ solution with /without voltage applied (two different methods of silver coating: galvanic displacement and electrodeposition)

Cyclic voltammetry (CV) tests were conducted in an aqueous solution of 0.4 mM HAuCl4 to determine the reduction peak of gold. The range of voltages tested was -0.5 V to 1 V. In this experiment, the reference electrode, counter electrode, and working electrode were Ag/AgCl, platinum, and PS-AgNW, respectively. Due to the oxidation peak of silver near 0.1 V, the reduction peak of gold was not observed. However, when using FTO as the working electrode, the reduction peak of gold was observed at E=0.5 V. To prevent silver oxidation during gold deposition, negative voltages were applied in the electrodeposition method. The applied voltages for nucleation potential and growth potential varied from -1.0 V for 1 second and -0.1 V for 20 to 100 seconds.

The graph of electrical current density during the electrodeposition process is shown for the PS-AgNW@Au(40) sample. The amount of charge transferred during the first second (nucleation voltage) was several times higher than the amount transferred in subsequent identical times. The total electrical charge transferred for different samples was calculated from the graph and plotted in the inset figure. Except for the first second (due to the higher voltage), the total charge transferred showed an almost linear relationship with time. The total charge transferred serves as an indicator of the thickness of the gold layer formed on the AgNWs.


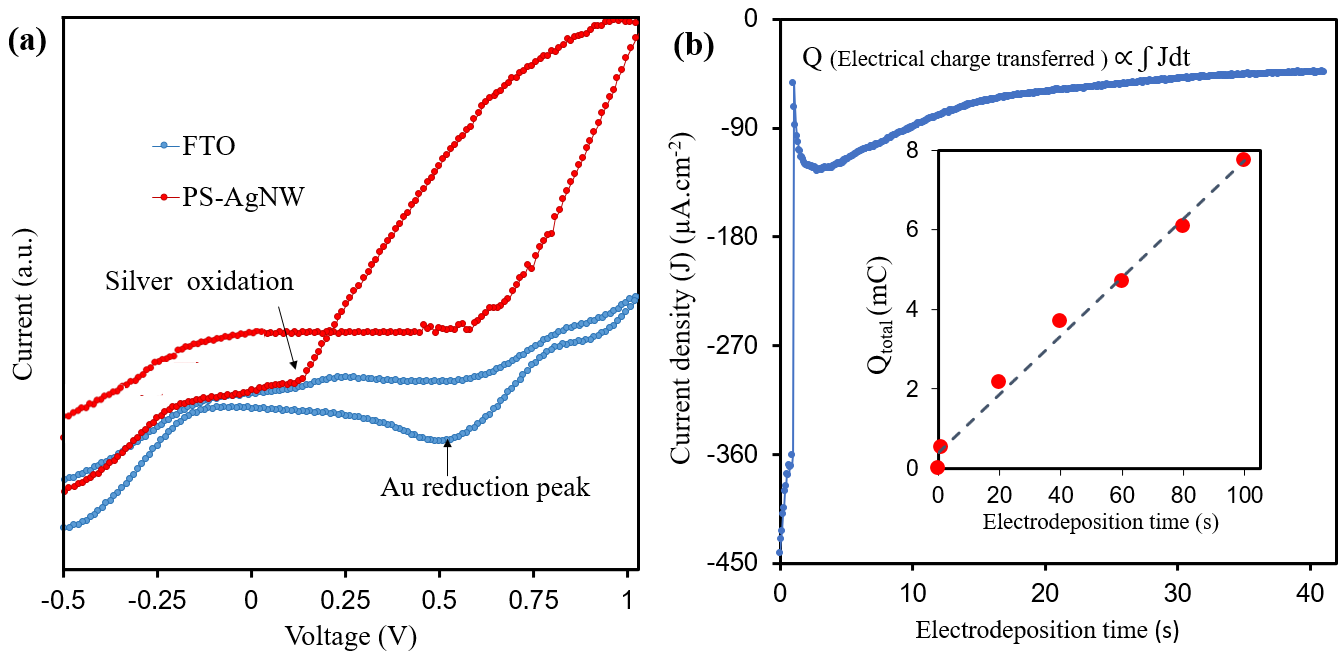


Figure S3. **a)** Plots of CV curves for FTO and PS-AgNW samples in 0.4 mM HAuCl_4_ aqueous solution. **b)** electrical current density During the electrodeposition process in the two-pulsed method (large pulse 1 s and small pulse 40 s) for a sample (PS-AgNW@Au(40)). Inset: the total electrical charge transferred vs. electrodeposition time (for 6 samples).

Table S2: The effect of gold electrodeposition on the sheet resistance of the samples

| R/R_0_ | | | |  |
| --- | --- | --- | --- | --- |
| Sample 4 | Sample 3 | Sample 2 | Sample 1 | Sample |
| 1.12 | 1.06 | 0.92 | 0.93 | PS-AgNW@Au(40) |
| 1.28 | 0.97 | 1.22 | 1.13 | PS-AgNW@Au(60) |
| 1.39 | 1.21 | 1.00 | 1.01 | PS-AgNW@Au(80) |
| - | - | 1.95 | 1.39 | PS-AgNW@Au(100) |

**EDX analysis**


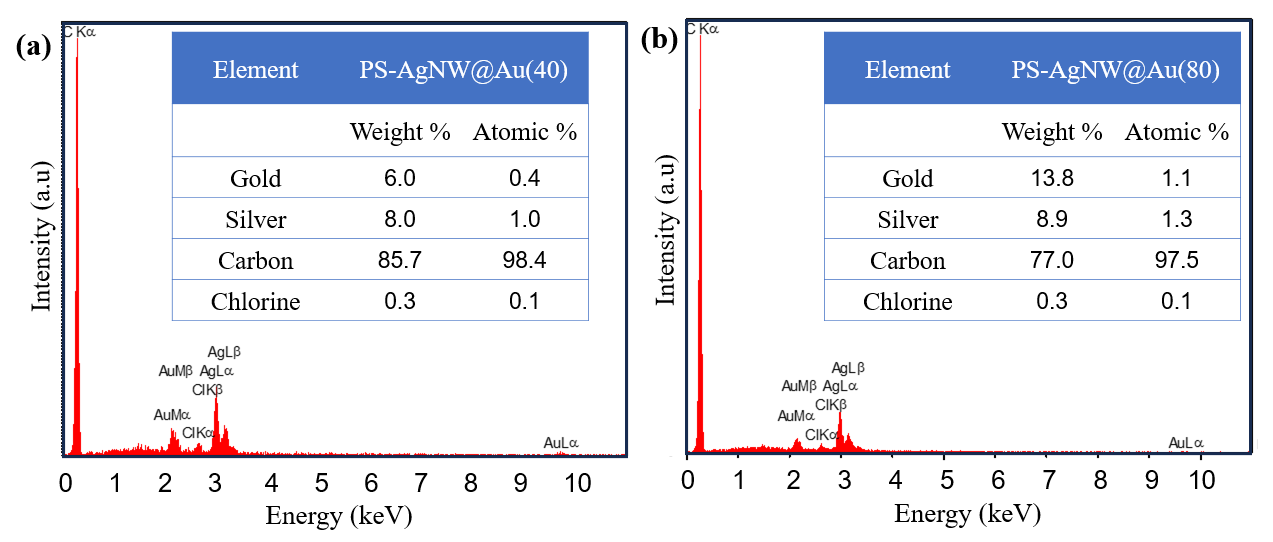


Figure S4. EDX analysis of **a)** PS-AgNW@Au(40) and **b)** PS-AgNW@Au(80)

**AFM results**


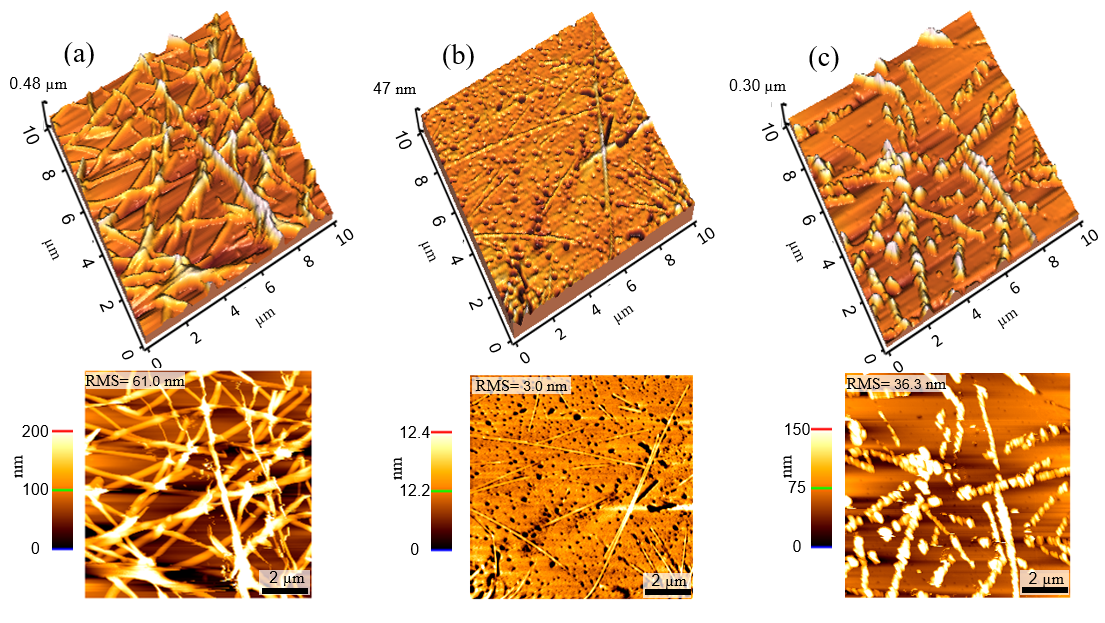


Figure S5. 3D and corresponding 2D AFM images of a) AgNW network on glass b) PS-AgNW c) PS-AgNW@Au(60)
